# The Molecular Diversity of Freshwater Picoeukaryotes Reveals High Occurrence of Putative Parasitoids in the Plankton

**DOI:** 10.1371/journal.pone.0002324

**Published:** 2008-06-11

**Authors:** Emilie Lefèvre, Balbine Roussel, Christian Amblard, Télesphore Sime-Ngando

**Affiliations:** LMGE, Laboratoire Microorganismes: Génome & Environnement, UMR CNRS 6023, Université Blaise Pascal (Clermont-Ferrand II), Aubière, France; Eawag, Switzerland

## Abstract

Eukaryotic microorganisms have been undersampled in biodiversity studies in freshwater environments. We present an original 18S rDNA survey of freshwater picoeukaryotes sampled during spring/summer 2005, complementing an earlier study conducted in autumn 2004 in Lake Pavin (France). These studies were designed to detect the small unidentified heterotrophic flagellates (HF, 0.6–5 µm) which are considered the main bacterivores in aquatic systems. Alveolates, Fungi and Stramenopiles represented 65% of the total diversity and differed from the dominant groups known from microscopic studies. Fungi and Telonemia taxa were restricted to the oxic zone which displayed two fold more operational taxonomic units (OTUs) than the oxycline. Temporal forcing also appeared as a driving force in the diversification within targeted organisms. Several sequences were not similar to those in databases and were considered as new or unsampled taxa, some of which may be typical of freshwater environments. Two taxa known from marine systems, the genera *Telonema* and *Amoebophrya*, were retrieved for the first time in our freshwater study. The analysis of potential trophic strategies displayed among the targeted HF highlighted the dominance of parasites and saprotrophs, and provided indications that these organisms have probably been wrongfully regarded as bacterivores in previous studies. A theoretical exercise based on a new ‘parasite/saprotroph-dominated HF hypothesis’ demonstrates that the inclusion of parasites and saprotrophs may increase the functional role of the microbial loop as a link for carbon flows in pelagic ecosystems. New interesting perspectives in aquatic microbial ecology are thus opened.

## Introduction

In aquatic environments, heterotrophic flagellates (HF) form a keystone group in the transfer of picoeukaryotic carbon to higher trophic levels [1], [2]. HF are regarded as an homogeneous functional group in the plankton, exclusively constituted of bacterivorous organisms. However, the lack of coupling between HF and bacteria in a range of marine and freshwater environments [3] questions the functional significance of this group. This has been tentatively explained by the facts that HF are not the only organisms that feed on bacteria and that they can consume viruses [4], [5] or colloïdal organic matter [6], [7]. Heterotrophic flagellates are not a phylogenetically coherent group since they include organisms from many branches of the eukaryotic tree of life [8]. Most of the studies that have attempted to assess the taxonomic composition of natural HF communities were exclusively conducted using conventional microscopic methods ([9]–[14]. However, because HF species display very few distinctive morphological features, the assessment of the taxonomic composition of natural assemblages remains a difficult task. This is especially true for the smallest HF which are usually classified as ‘small unidentified’ and represent the major part of the HF community [15].

An alternative to the conventional microscopic techniques is the use of molecular methods. Numerous recent picoeukaryotic environmental rDNA surveys, mostly conducted in marine systems [16]–[22], have revealed an unexpected high diversity within lineages that contain HF forms. In freshwater systems, there is a lack of sequences in the available nucleotide databases for the assessment of the community composition of cultured and uncultured heterotrophic flagellates [23]–[26]. Such an assessment is of significance in elucidating the functional role of HF in aquatic microbial food webs and the related biogeochemical cycles, because HF display diverse trophic strategies others than the sole phagotrophy (e.g. [27]). The aims of this study include (i) the examination of the phylogenetic composition of small eukaryotic cells (<5 µm) in a deep meromictic freshwater lake. This study was conducted in spring/summer 2005 and was designed as a seasonal complement to a similar companion study we conducted in the same lake in autumn 2004 [23]. Comparisons between the two studies also have allowed us to (ii) estimate the temporal dynamics in the diversity of picoeukaryotes, and (iii) discuss the trophic strategies displayed within the small targeted HF assemblages, and their potential ecological and biogeochemical implications.

## Materials and Methods

**Figure 1:**
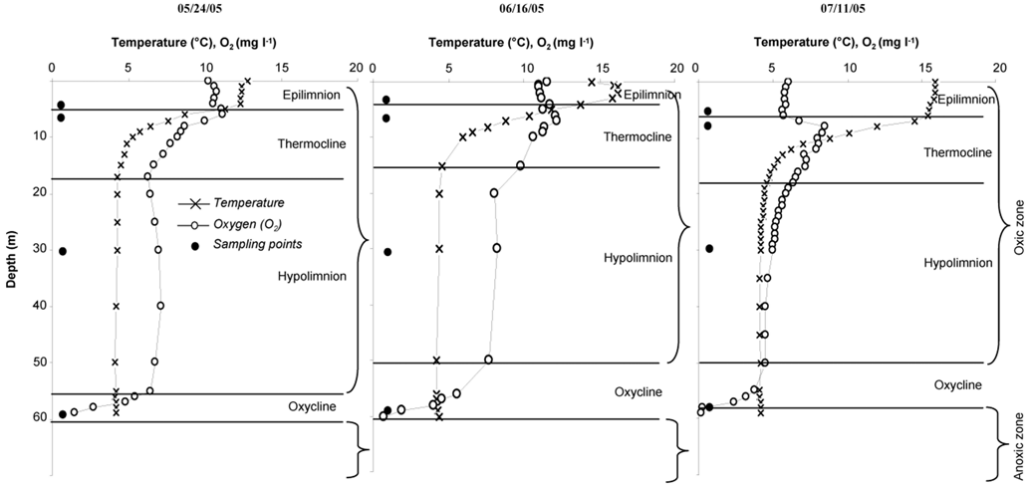
Vertical profiles of temperature and oxygen concentration measured at the three sampling dates in Lake Pavin.

Lake Pavin is a meromictic, dimictic and oligo-mesotrophic lake situated in the Massif Central of France (02°53′12″E, 45°29′41″N). It is a deep volcanic mountain lake characterized by a low drainage basin area (50×10^4^ m^2^). This site offers a unique environment with low human influences and high annual reproducibility of seasonal dynamics in the water column [23]. Three different depths were sampled for the oxic layer of the lake [i.e. 5 m (epilimnion), 9–10 m (metalimnion), and 30 m (hypolimnion)], and one depth (59 m) for the oxycline at three occasions (May, 24; June, 16 and July, 11 2005) (Fig. 1). Vertical profiles of temperature and dissolved oxygen were obtained for each sampling date. Cells of interest (<5 µm) were collected on 0.6 µm-pore polycarbonate filters and stored at −80°C until DNA extraction.

Following cell lyses with proteinase K, total community DNA was extracted by a classical CTAB protocol. The entire SSU rDNA gene was amplified using the eukaryotic primers 1f and 1520r and cloned using the TOPO-TA cloning kit (Invitrogen). For each sampling dates, two genetic libraries where constructed: one for the oxic zone (obtained by pooling individual PCR products from the three oxic depths sampled), and one for the oxycline, i.e. a total of six libraries over three sampling dates. Genetic polymorphism was then assessed by analyzing several clones by restriction fragment length polymorphism (RFLP) using the restriction enzyme *Hae*III. Clones showing the same RFLP patterns were grouped and considered to belong to the same operational taxonomic unit (OTU). One representative of each OTU was sequenced from plasmid products by the MWG Biotech services. Sequences were aligned using the alignment tool of the ARB package (http://www.arb-home.de/) and Bioedit software (http://www.mbio.ncsu.edu/BioEdit/bioedit). Phylogeny was inferred both by the Neigbhor Joining and the Bayesian methods, but only the tree generated by the Bayesian method was shown. Detailed experimental procedures are provided in Lefèvre et al. [23]. Nucleotide sequences obtained in this study have been deposited in Genbank under Accession Numbers EU162617-EU162648.

## Results and Discussion

### Temperature and dissolved oxygen

Temperature profiles indicate that our sampling period corresponded to the thermal stratification of the lake, with a relatively constant temperature in the epilimnion. Oxygen concentration was at 5–12 mg l^−1^ in the epilimnion and increased slightly in the first upper meters of the metalimnion, as a result of the intense activity of photosynthetic organisms at these depths [23]. Oxygen content decreased in the metalimnion to reach 4–7 mg l^−1^ throughout the hypolimnion. Values then dropped in the oxycline layer to <1 mg l^−1^ at 60 m depth (Fig. 1).

### Overall diversity estimates

Among the 6 libraries constructed, rarefaction curves [28] did not reach saturation level except for one library, the oxycline in June (Figure S1). The high coverage value reached for this library (94.2%, Table S1) indicates that this community was well sampled and that most of the targeted diversity was detected [28]. Although the sampling effort was larger for this study (484 clones analysed by RFLP) compared to the survey we conducted in autumn 2004 (252 clones analysed [23]), the coverage values calculated for the two studies were roughly the same, ranging from 77 to 94.6% (average 88%) in spring/summer 2005 and from 76.3 to 92.1% (average 83%) in autumn 2004. This supports the conclusion that the picoeukaryotic diversity in Lake Pavin was higher in our spring/summer than in autumnal samples [28]. In general, the oxic zone displayed twofold more OTUs than the oxycline (49 and 26 OTUs, respectively) for a relatively similar amount of clones analysed (277 and 207, respectively). The two sampling zones shared about 30% of OTUs (Table S1), indicating the ability of some picoeukaryotes to colonize the whole mixolimnic layer of the lake, regardless of oxygen constraints. However, it is likely that part of these organisms originated from sinking processes and, probably, was not a physiologically active fraction in the oxycline. Indeed, many unicellular microorganisms, especially prasites, are known to produce resting forms (in response to harsh environmental conditions) that can sink through the water column [29]. In addition, free DNA from organisms that do not actually live in the oxycline could be present in this layer [20], [30].

### Gross genetic affiliations of sequences

The taxonomic distribution of our sequences comprised 8 major eukaryotic phyla (Figure S2). When OTU redundancies in the different libraries were excluded, three taxonomic groups represented 65.4% of the detected diversity: Alveolates (32.7%), Fungi (19%), and Stramenopiles (13.7%). The five other groups retrieved generally represented <10% of the total OTUs, comprising Cryptomonads, Chlorophyceae, Cercozoa, Telonemia, and Choanozoa. Among Alveolates, 75% of our sequences belonged to lineages known to contain members that produce small colourless flagellates with two unequal flagella during the motile stage of their life cycle, i.e. Perkinsea and Dinophyceae. Among the kingdom Fungi, 75% of the sequences were related to the parasitic or saprotrophic Chytridiomycota which produce small uniflagellated cells (zoospores) during their life cycle. Among Stramenopiles, 75% of our sequences were affiliated to the small heterotrophic biflagellated Bicosoecids and uniflagellated Hyphochytrids. The major part of the sequences retrieved thus fell into ecologically important taxonomic groups that are known to contain small heterotrophic flagellated forms.

All detected taxonomic groups but Chlorophyceae and Fungi were represented in both the oxic zone and in the oxycline libraries (Figure S2). Chlorophyceae were restricted to the oxic zone where they can participate in the oxygen peak production observed in the upper part of the metalimnion. Fungal phylotypes contributed substantially to the picoeukaryotic diversity within our oxic libraries and the majority belonged to the fungal class Chytridiomycota. None of our fungal sequences were affiliated to the order Neocallimastigales or the order Blastocladiales which contain species that have been cultured under strict anaerobic conditions [31].

### Phylogenetic tree topologies

**Figure 2:**
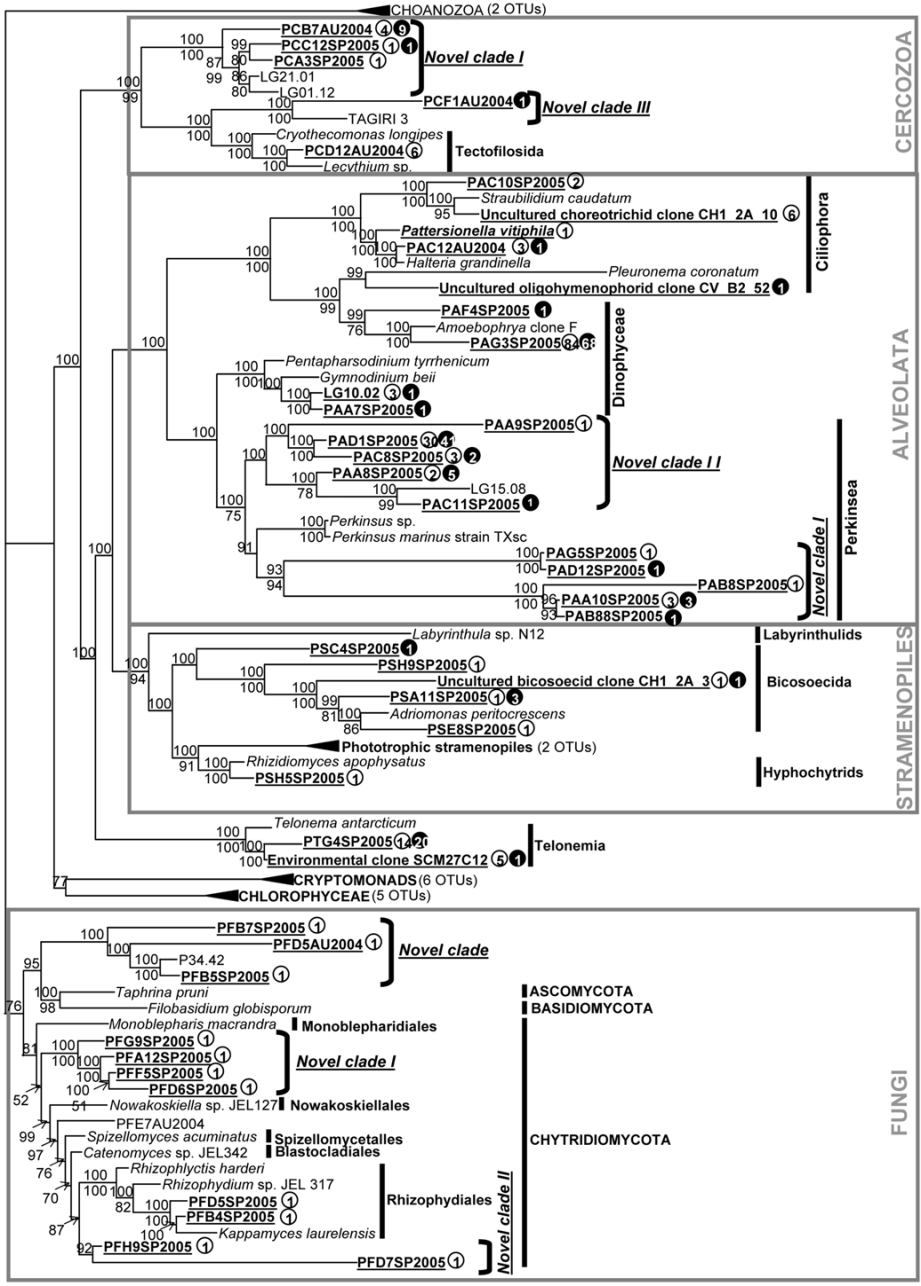
Bayesian phylogenetic tree of 18S rRNA gene obtained from the Lake Pavin. The Bayesian unrooted tree was inferred from an alignment of 88 sequences with a character sampling of 1511 nucleotides. The origin of phylotypes from lake Pavin and the number of clones are indicated after each of our underlined environmental sequences. A white circle symbol corresponds to the oxic zone and a black one to the oxycline. The numbers (given as percentages) above and below the nodes correspond to Bayesian Posterior Probabilities and bootstrap values calculated using the Neighbor-Joining method, respectively.

Sequences retrieved in this study were all affiliated to known lineages, contrasting with studies conducted in permanently anoxic or other extreme environments where novel eukaryotic kingdom-level lineages were recorded [32]–[35]. Interestingly, 83% of our sequences were not related to any previously published sequences, including 23 sequences that belonged to novel clades (i.e. currently containing no cultured representatives). This suggests that the eukaryotic microbial diversity is still poorly described, primarily in freshwater environments. Because this study was designed to detect taxonomic diversity of ‘small unidentified’ HF, taxonomic groups known to contain small HF forms (i.e. Alveolates, Fungi, Stramenopiles, Cercozoa and Telonemia) were highlighted in the phylogenetic tree (Fig. 2).

#### Alveolates

The clone libraries constructed yielded 19 Alveolate sequences, 10 of which were closely related to Perkinsids, 4 to Dinophyceae and 5 to Ciliophora (Fig. 2). Perkinsozoa affiliated sequences were divided in two distinct novel clades. 5 sequences formed the relatively well supported novel clade I which branched as a sister group to the genus *Perkinsus*. Another group of 5 sequences grouped together with the Lake George environmental sequence LG15.08 [26] to form another very well supported clade II, branched at the base of the novel clade I and of the *Perkinsus* species with high support value. All members of the Perkinsozoa lineage produce free-motile biflagellate zoospores of 2–7 µm in diameter that do not display distinctive morphological characteristics [36], [37]. This could explain why perkinsids have never been observed in studies that used microscopic methods, suggesting that the zoosporic form of these perkinsid organisms could have been miscounted as phagotrophic flagellates in previous studies. Among the 4 sequences of Dinophycean Alveolates, 2 were related to the genus *Amoebophrya* which is commonly detected in picoeukaryotic marine rDNA surveys [17], [18], [20], [32], [34]. To the best of our knowledge, this is the first time that *Amoebophrya*-like phylotypes are detected in a freshwater lake. The life cycle of *Amoebophrya* species include a biflagellate dispersal stage of 5 µm in size, the dinospore [38]. This contrasts with the current idea that categorizes dinoflagellates as large HF (>15 µm) [39], and demonstrates that our knowledge of the diversity of small dinoflagellates is deficient (40), primarily in freshwaters.

#### Fungi

Among the Fungi-affiliated sequences (19% of the total OTUs), 4 affiliated to a novel clade described in previous studies [23], [25], and the others to Chytridiomycota. This class of Fungi is characterized by the production of microscopic uniflagellated free-swimming zoospores in their reproductive life stage. Identity of our chytridiomycete sequences to previously recorded sequences in the Genbank was not found, indicating that the diversity of this group is still very poorly known. During the present study, only two sequences clustered within the order of Rhizophydiales, Letcher, ord. nov. [41]. Based on zoospore ultrastructure and molecular characteristics, this new order encompasses 4 genera previously classified within the order of Chytridiales, including the genus *Rhizophidium* which was previously subclassified within the “Rhizophidium clade” [42]. 5 other environmental sequences clustered together (with high support) to form an early divergent novel clade within the Chytridiomycota. An additional group of 2 fast-diverging sequences, as attested by the long-branches they formed in the tree topology, also constituted a novel clade within Chytridiomycota (Fig. 2). These two novel clades seem to have an order taxonomic level position and may constitute non described orders within Chytridiomycota. Moreover, it is noteworthy that the high diversity of Chytridiomicota-affiliated sequences reported in our studies has never been detected before, corroborating the idea that freshwaters are really undersampled in terms of eukaryotic microbial diversity.

#### Stramenopiles

Among the 8 Stramenopile sequences detected, 2 were related to the phototrophic lineages of Bacillariophyceae and Chrysophyceae. The remaining sequences were affiliated to 2 heterotrophic phyla, the ‘Fungi-like’ Hyphochytrids and the biflagellate Bicosoecids. Hyphochytrids were represented by only one sequence that was robustly branched to *Rhizidiomyces apophysatus*. This species possess many morphological and ecological similarities with chytrids and produce cells with single anteriorly directed tinsel flagella during one stage of their life cycle. The presence of bicosoecid sequences in our libraries was not surprising because they were reported to dominate HF community in June and July in Lake Pavin [9].

#### Cercozoa

5 phylotypes grouped within the Cercozoa phylum with high supports. Among them, 3 were already detected in autumn [23], including 1 sequence that belonged to the freshwater order of Tectofilosida which comprises small heterotrophic amoeboid flagellates. The two new phylotypes clustered as novel clade I of Cercozoa (Fig. 2) that seems typical of freshwater systems as attested by the presence of two Lake George sequences in this group [26]. Bass and Cavalier-Smith [43] have conducted a cercozoan environmental rDNA analysis in a range of different habitats and have highlighted 9 novel clades, each of which comprised sequences from only one habitat or sampling site. This suggests that some cercozoan clusters may represent geographycally or ecologically niche-restricted taxa.

#### Telonemia

2 of our environmental sequences strongly affiliated with the genus *Telonema* which, to date, comprises only two species described as phagotrophic free-living biflagellated organisms of 6–8 µm in length [44]. The genus *Telonema* is one of the most widely reported colourless flagellates in marine ecosystems [45]. More recently, a phylogenetic study has shown that marine environmental sequences grouped together with the two known *Telonema* species to form an early diverging monophyletic lineage considered as Telonemia phylum [44]. To the best of our knowledge, this is the first time that sequences related to the genus *Telonema* are retrieved from a freshwater system.

### Temporal dynamics in the HF diversity

**Figure 3:**
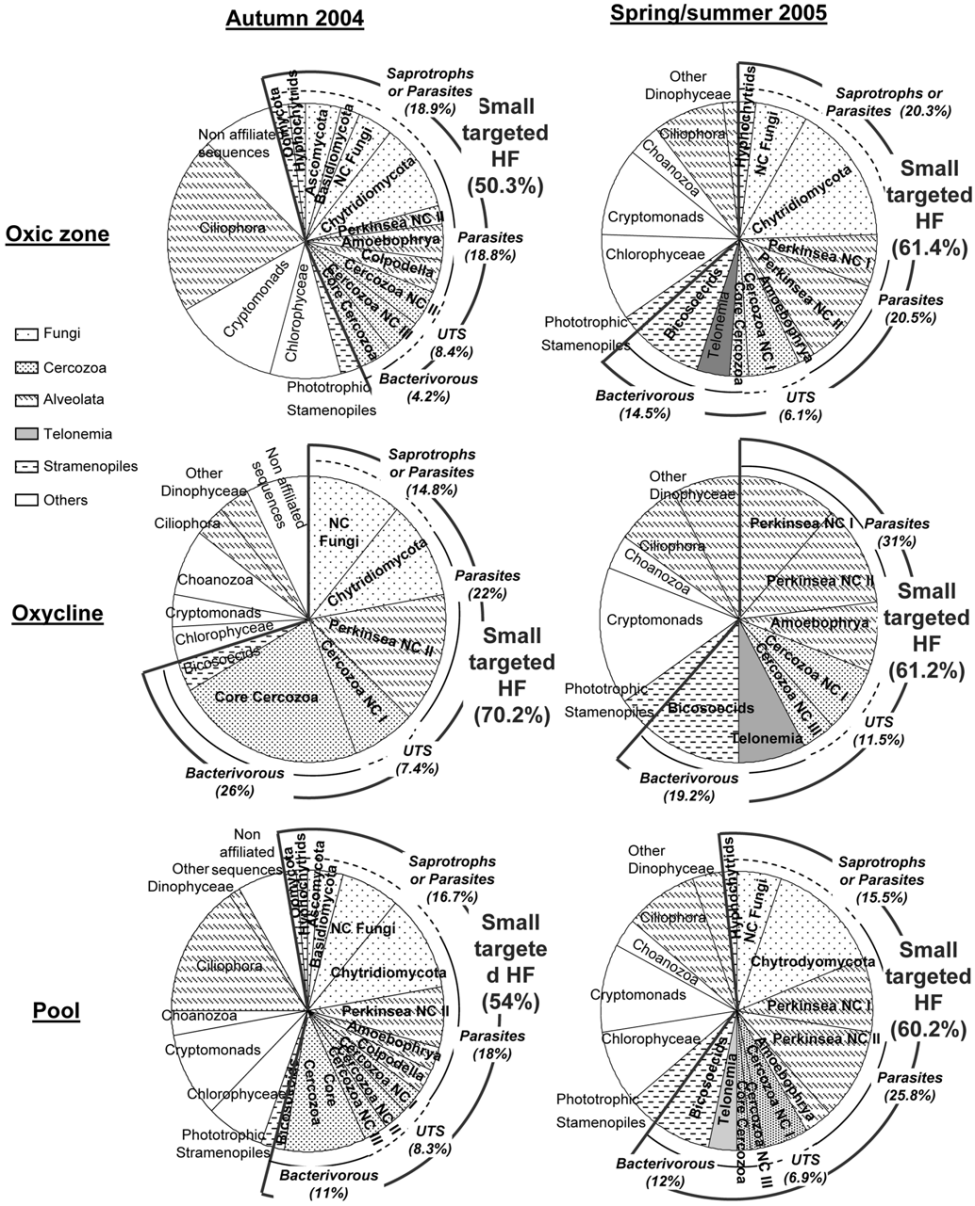
Relative abundances of OTUs retrieved from the picoplanktonic fraction of the oxic zone and the oxycline of the Lake Pavin during autumn 2004 (23) and spring/summer 2005 (this study). For each graph, the relative abundances of OTUs corresponding to different trophic strategies displayed within the five taxonomic groups containing the targeted small flagellates are proposed. For novel clades (i.e. with no cultured representatives; NC), trophic strategies were not attributed.

A similar study was previously conducted in the same depths in Lake Pavin but during autumn 2004 [23], providing an opportunity to estimate the temporal dynamics in the diversity of picoeukaryotes (cf. Fig. 3). Only 5 of the 58 sequences were common to autumn 2004 and spring/summer 2005, indicating ‘seasonal’ or interannual dynamics within the diversity of the targeted picoeukaryotes. Telonemia phylum occured only in spring/summer, together with the other major taxonomic phyla (i.e. Alveolates, Fungi, Stramenopiles, Cercozoa) detected in the two consecutive studies. Alveolates and Fungi roughly represented the same proportions in autunm (30% and 23%, respectively) and in the present study (32.7% and 19%), while the diversity of Cercozoa significantly decreased (from 19% in autumn to 8.6% in spring/summer).

Compared to Alveolates and Fungi, the dynamics of the diversity within Stramenopiles and particularly within Cercozoa exhibited weak ‘seasonality’. The relative importance of heterotrophic Stramenopile lineages increased with time from 25% (autumn) to 75% (spring/summer), probably due to the summer development of Bicosecids in Lake Pavin [9]. Within the total diversity of Alveolates, the relative importance of Perkinsids increased from autumn (24%) to spring/summer (50%). The presence of ciliates in our picoplanktonic samples could be related to the passage of some smallest individuals (15–20 µm in size) through the 5 µm pore size membranes used [46], but was probably the result of cell breakage during filtrations [23]. Within Fungi, the relative diversity of Chytridiomycota dominated, increasing from 50% in autumn to more than 73% of the total fungal diversity in spring/summer. In addition, there was no shared sequence between the two sampling periods, suggesting an overall high diversity and a marked ‘seasonality’ in this group of organisms although the two studies were conducted in different years.

### Trophic strategies among the targeted HF

#### Overall comparisons

(i) Based on the phylogenetic affiliation of the sequences recovered from the autumn and spring/summer samples, (ii) and based on the assumption that trophic behaviors ascribed to the retrieved phylotypes are the same displayed by their closely affiliated known representants, the potential trophic strategies displayed among the targeted small heterotrophic flagellate communities were estimated as follows: 19–31% for parasitism, 15–20% for saprotrophy and (or) parasites (see below), and 4–26% for bacterivory. In addition, there were 6 to 12% novel clades of cercozoan-affiliated sequences for which we were not able to classify. Sequences belonging to the non-targeted phylotypes, corresponding to autotrophs, ciliates, and large flagellates, represented 30–50% of the total diversity (Fig. 3). These groups were excluded from comparisons. The contributions of the three trophic strategies within the targeted small HF are thus considered as underestimates.

#### Parasitism

Among Alveolates, a substantial part of our detected sequences were closely related to the genera *Perkinsus, Amoebophrya,* and *Colpodella* which are known as strict parasites in aquatic systems. Species belonging to the genus *Perkinsus* are important endoparasites of oysters [47], [48] and fishes [49]. They are phylogenetically affiliated to the Perkinsea lineage together with *Parvilucifera infectans,* an endoparasite of more than 25 species of dinoflagellates [50], and with C*ryptophagus subtilis*, an endoparasite reported in *Chilomonas paramecium* [37]. *Amoebophrya* species were also found to infect more than 35 species of dinoflagellates and other planktonic microorganisms, including ciliates [50]. *Amoebophrya* species, which are mainly known as marine endoparasite in red-tide dinoflagellate blooms [51], were intensively studied in oceans where they display a worldwide distribution [50]. Species of the genus C*olpodella* are small (<10 µm in size) predators of other free-living protists larger than themselves, such as *Bodo caudatus*, *Spumella* sp. and *Chilomonas paramecium* [37], [52]. They penetrate host cells using their apical rostrum and aspirate the host cytoplasm within a few minutes [37], [53], a trophic strategy that we associate with parasitism. Others potential parasites retrieved during our studies were the fungal members of Chytridiomycota. The Rhizophydiale order of Chytridiomycota indeed occurs primarily as phytoplankton parasites in aquatic ecosystems [41]. This is supported by the observation that a substantial fraction of phytoplanktonic species in Lake Pavin and other lakes in the vicinity are intensively parasitized by chytrids throughout the year (S. Rasconi et al., pers. com.).

#### Potential Saprotrophy

The kingdom of Fungi exclusively comprised parasites and saprotrophs. Within Chytrids, the order of Monoblepharidiales has been described from saprotrophic species only, but they were not sampled in our studies. The other Chytridiomcota orders comprise both saprotrophic and parasitic representants. Although the definitions of these terms seem clear, the frontier between saprotrophy and parasitism may be ambiguous. For example, Alster and Zohary [54] recently have observed that the chytrid *Phlyctochytrium* sp. mostly infects stressed or dead cells of a bloom-forming dinoflagellate species in Lake Kinneret, Israel. These authors then concluded that this Chytrid was saprotroph or possibly facultative parasite, although the infection of healthy dividing host cells is considered a general rule in chytrid parasitism [55]. It was thus not possible in our estimation to separate saprotrophs from parasites others than Rhizophidiales (Fig. 3). The same applies to the so-called ‘Fungi-like’ Stramenopiles (i.e. Hyphochytrids and Oomycetes) which typically exhibit similar life styles as Chytridiomycetes [56].

#### Bacterivory

In our surveys, the small HF sequences belonging to typical bacterivores were those from the Stramenopiles group of Bicosoecid species which are abundant components in marine and freshwater pelagic systems [9]. An additional typical bacterivorous HF group, recently defined as the new protistan phylum Telonemia [44], was aslo detected in our spring/summer survey. The phylum is known from two species isolated in marine systems and described as small (<10 µm in size) bacterivorous flagellates [45].

#### Unresolved trophic strategy (UTS group)

In our two studies, sequences affiliated to the ‘core cercozoa’, (*sensu* Nikolaev and coauthors, [57]) were all closely related to the genera *Lecythium* and *Cercomonas*. These two genera encompass small amoeboid flagellated species (<10 µm in size) known as bacterial feeders in various terrestrial and aquatic ecosystems [57], [58]. Although most heterotrophic Cercozoa are bacterivorous, this phylum also contains predatory species such as *Cryothecomonas longipes* and *C. aestivalis* that phagocytize the cytoplasm of various host diatoms in marine systems using pseudopodia [59], [60]. Other species such as *Proleptomonas faecicola* are known as saprotrophs [61] even though they are restricted to soil habitats. Because among Cercozoa all the trophic strategies are displayed, they were pooled in the UST group (Fig. 3).

### Potential ecological implications of the high occurrence of parasitoids and saprotrophs

**Figure 4:**
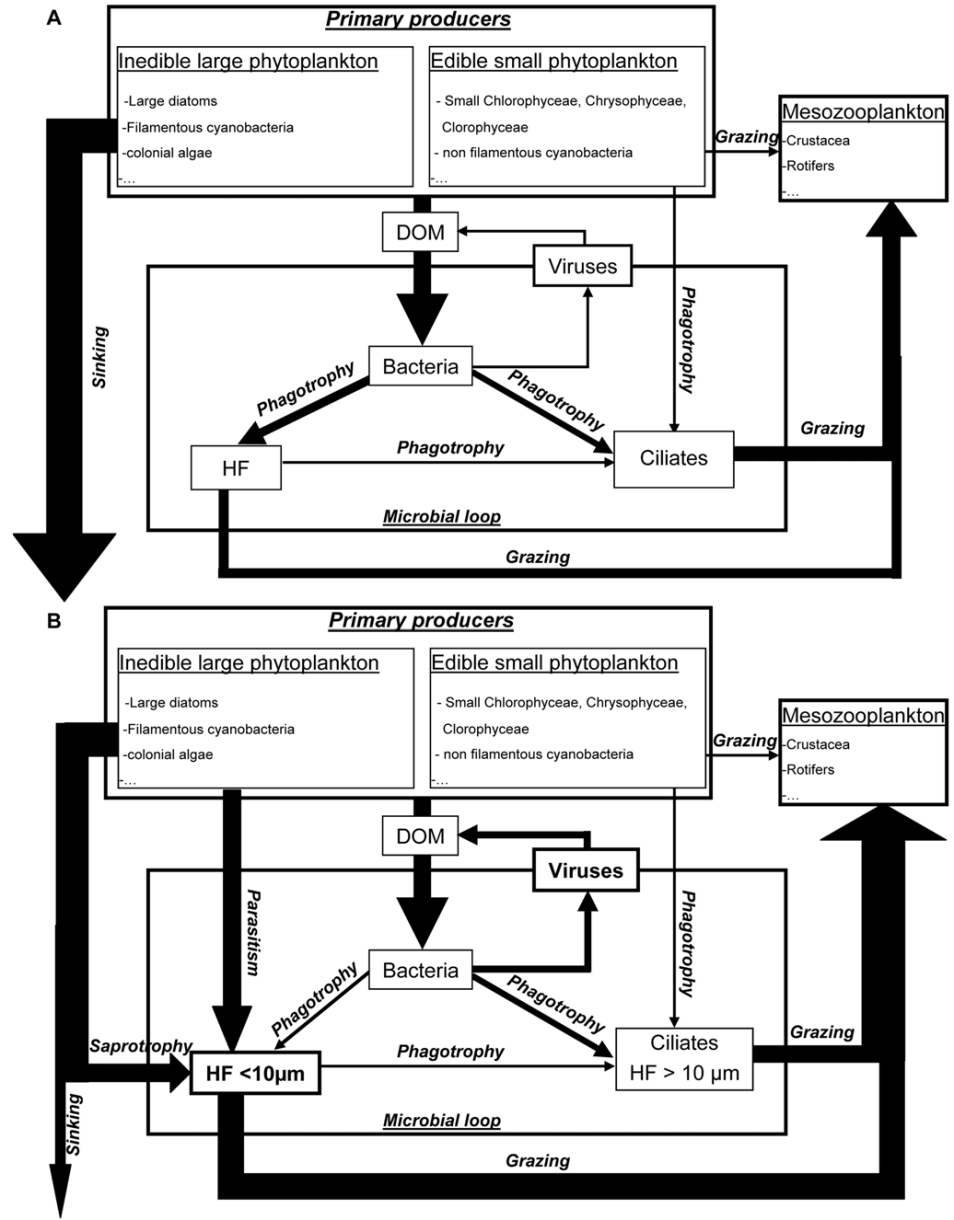
Conceptual models of carbon flows between primary producers, grazing zooplankton, and microorganisms in pelagic ecosystems. (A) Classical model where heterotrophic flagellates (HF) are considered as a unique functional community of bacterial grazers, i.e. *the ‘bacterivore-dominated HF hypothesis’*. A substancial part of the primary production is not grazed by mesozooplankton but either used by the microbial loop or lost by sinking. (B) Proposed conceptual model based on the new *‘parasite/saprotroph-dominated HF hypothesis’*. In this model, heterotrophic flagellates are shared into HF <10 µm and HF >10 µm, the former comprising the whole HF trophic strategies (i.e. parasitism, saprotroph, and bacterivory) that are responsible for the incorporation of sinking matter into microbial food webs and its tranfer to the higher trophic levels. The differences in the thickness of the arrows represent the preferential carbon pathways, although no quantitative data are yet avalaible.

The analysis of potential trophic strategies has indicated that strict bacterivorous picoflagellates were represented by only few taxa in our studies, and were less diverse than parasitic and (or) saprotrophic forms. For the two studies conducted in autumn and in spring/summer and when pooling the data from the two sampling zones, the minimal contributions of the diffetent trophic strategies to the targeted HF diversity indeed were at about 12, 22, and 16%, for the groups of bacterivores, parasites and saprotrophs/parasites, respectively (Fig. 3). Although these considerations are based on phylogenetic affiliation of the retrieved sequences, microscopic observations of parasitized phytoplanktonic species and evidences from the literature suggest that, in contrary to the commonly accepted idea, small heterotrophic flagellate communities are not exclusively bacterivores in aquatic systems. The high occurrence of sequences affiliated to parasitic and saprotrophic HF-containing lineages could be of paramount importance, as the potential ecological implications can significantly change our conception of the carbon flows in aquatic microbial food webs (Fig. 4).

The last 30 years have revealed major conceptual advances in pelagic microbial ecology – arguably the greatest in environmental sciences. Two major concepts have emerged: the ‘microbial loop’ where nutrients are recycled through bacteria-grazer interactions, and the ‘microbial food web’ that includes the relationships between heterotrophic and autotrophic microorganisms and its relationships to biogeochemical cycles [1], [62]. In the simplified concept of microbial loop, a substantial fraction of carbon fixed by primary producers is released as dissolved organic carbon that is transferred back to the classical food chain (Phytoplankton → metazooplakton → fishes) *via* a 2–3 steps microbial food chain (i.e. microbial loop), where bacteria and protistan flagellates and ciliates are key intermediates (Fig. 4A). A high number of trophic levels in the microbial loop together with the low assimilation efficiency have stimulated an extensive debate in the literature concerning the importance of the microbial loop as a link or as a sink of carbon in pelagic systems [63]–[65]. Large phytoplankton cells or colonies which are inedible for metazooplankton can sink through the water column and be lost from the system as a huge amount of carbon. In this particular scenario which most likely dominates the productivity of the world pelagic system (i.e. phytoplankton bloom situations), the efficiency of the microbial loop as a link of carbon to the upper trophic levels is largely weakened. This is in the context of our present conception that the small heterotrophic flagellates are the major and exclusively bacterivores, i.e. the ‘bacterivore-dominated HF hypothesis’ (Fig. 4A).

In the same scenario but under the ‘parasite/saprotroph-dominated HF hypothesis’ that includes the whole trophic strategies displayed within small heterotrophic flagellates, the functional response of the microbial loop as a link may be significantly enhanced. The major ecological implication could be the increase of the system productivity from the energy upwelled by small HF whole activities (i.e. bacterivory, parasitism, saprotrophy), even if the system respiration may also increased *via* the saprotrophic stimulation of the viral loop (Fig. 4B). Although our proposed ‘parasite/saprotroph-dominated HF hypothesis’ needs to be properly tested with quantitative data, the hypothesis is supported by major lines of evidence. The first line is provided in both our autumnal and spring/summer environmental rDNA surveys of picoeukaryote diversity, where we highlight that a significant fraction of the overall diversity of heterotrophic picoflagellates is probably composed of non bacterivorous organisms, but may be flagellates or flagellated stages of parasitic or saprotrophic microorganisms. In our lake, unidentified small flagellates averaged 70% of the seasonal abundances of HF [9]. In other lakes over the world, the contributions of undetermined small HF (<10 µm) are also significantly high, ranging from 10% in Lake Piburger to 89% in Lake Traunsee, Austria (Table S2). In terms of carbon biomass, these contributions remain substantial (Table S2) and can reach 55 to 90% as shown from a study conducted in 55 different lakes in Germany [10].

Another line of evidence supporting our proposed new hypothesis comes from recent studies that have clearly highlighted that many inedible phytoplankton species are intensively parasitized by fungal chytrids in numerous freshwater systems [55], [66]. More interestingly, Kagami *et al.* [67], [68] have demonstrated that the small flagellated zoospores produced by parasitic chytrids from their phytoplankton hosts are efficiently grazed by crustacean zooplankton, as also noted previously for the *Amoebophrya* flagellated dinospores [38]. This supports the idea that in addition to returning energy lost as phytoplanktonic exudates to higher trophic level, the microbial loop also returns a large amount of energy lost as sinking phytoplankton cells, primarily during blooms. Because we have observed that parasitized phytoplankton cells are common and quantitatively important in several temperate lakes (Rasconi et al., pers. com.) and based on the above observations, we propose that the dominant phytoplankton communities which are susceptible to eukaryotic parasites or saprotrophs are host to thriving populations of overlooked small heterotrophic flagellates, whose trophic activities may increase the functional role of the microbial loop as a link for carbon flows in pelagic ecosystems.

## Supporting Information

Table S1 Results of RFLP analysis of the four picoeukaryotic 18S rDNA libraries generated from Lake Pavin, spring/summer 2005.
(0.05 MB DOC)

Table S2 Relative abundance and/or biomass of unidentified heterotrophic nanoflagellates (HNF) in a range of freshwater lakes differing in their trophic status.
(0.05 MB DOC)

Figure S1 Rarefaction curves for six Lake Pavin libraries, spring/summer 2005. Clones were grouped into OTUs at a level of sequence similarity of ≥97%
(0.07 MB DOC)

Figure S2 Relative abundances of OTUs within the 8 phylogenetic groups represented in our libraries from Lake Pavin, spring/summer 2005. Taxonomic subgroups are defined for the major taxa. The numerical abundances (i.e. % of total OTUs) are given for each subgroup
(0.31 MB DOC)
