# Supplementary material for: The Molecular Diversity of Freshwater Picoeukaryotes Reveals High Occurrence of Putative Parasitoids in the Plankton
Source: PLoS One. 2008 Jun 11;3(6):e2324. doi: 10.1371/journal.pone.0002324 (PMC2396521; doi:10.1371/journal.pone.0002324)
Supplement: Table S1 — Results of RFLP analysis of the four picoeukaryotic 18S rDNA libraries generated from Lake Pavin, spring/summer 2005. (0.05 MB DOC) [file pone.0002324.s001.doc]

**Table S1.**  Results of RFLP analysis of the four picoeukaryotic 18S rDNA libraries generated from

Lake Pavin, spring/summer 2005.

| Zones | Libraries | No. of OTUs (clones) per library | No. of OTUs unique to library | No. Of OTUs shared to library | Coverage (%) | Estimated no. of OTUs S_chao1_ | No. of OTUs (clones) per zone | No. of OTUs unique to zone | No. of OTUs shared with zone |
| --- | --- | --- | --- | --- | --- | --- | --- | --- | --- |
| Oxic zone | Oxic zone 05/24/05 | 23 (61) | 16 | 7 | 77 | 72 | 49 (277) | 32 (65.5%) | 17 (28.96*) |
|  | Oxic zone 06/16/05 | 15 (50) | 7 | 8 | 86 | 21.1 |  |  |  |
|  | Oxic zone 07/11/05 | 25 (166) | 8 | 17 | 94.6 | 33.1 |  |  |  |
| Oxycline | Oxycline 05/24/05 | 18 (51) | 3 | 15 | 82.4 | 31.5 | 26 (207) | 9 (34.6%) |  |
|  | Oxycline 06/16/05 | 9 (69) | 1 | 8 | 94.2 | 15 |  |  |  |
|  | Oxycline 07/11/05 | 15 (87) | 4 | 11 | 92 | 27.3 |  |  |  |

^a^ Estimated number of the shared OTUs based on the sample coverage approach (heterogeneous model) proposed in Chao *et al.* (2000). The non parametric Schao1 estimator is currently considered to be the best richness estimator for microbial clone libraries (Hughes et al. 2001)

**References**

Chao A, Hwang WH, Chen YC, Kuo CY (2000) *Statistica Sinica* 10: 227-246.

Hughes JB, Hellmann JJ, Ricketts TH, Bohannan BJM (2001) *Appl Environ Microbiol* 67: 4399-4406.
