# Supplementary material for: The Molecular Diversity of Freshwater Picoeukaryotes Reveals High Occurrence of Putative Parasitoids in the Plankton
Source: PLoS One. 2008 Jun 11;3(6):e2324. doi: 10.1371/journal.pone.0002324 (PMC2396521; doi:10.1371/journal.pone.0002324)
Supplement: Table S2 — Relative abundance and/or biomass of unidentified heterotrophic nanoflagellates (HNF) in a range of freshwater lakes differing in their trophic status. (0.05 MB DOC) [file pone.0002324.s002.doc]

**Table S2.**  Relative abundance and/or biomass of unidentified heterotrophic nanoflagellates (HNF) in a range of freswater lakes differing in their trophic status.

**References**

Auer B, Arndt H (2001) *Freshw Biol* 46: 959-972.

Carrias JF, Quiblier-Lloberas C, Bourdier G (1998) *Freshw Biol* 39: 91-101.

Cleven E.-J, Weisse T (2001) *Aquat Microb Ecol* 23: 147-161.

Comte J, Jacquet S, Viboud S, Fontvieille D, Millery A, Paolini G, Domaizon I (2004) *Microb Ecol* 52: 72-89.

Domaizon I, Viboud S, Fontvieille D (2003) *FEMS microbiol Ecol* 46: 317-329.

Pernthaler J, Sattler B, Simek K, Schwarzenbacher A, Psenner R (1996) *Aquat Microb Ecol* 10: 255-263

Sonntag B, Posch T, Klammer S, Teubner K, Psenner R (2006) *Aquat Microb Ecol* 43: 193-207.
