# Supplementary figures and images for: The Molecular Diversity of Freshwater Picoeukaryotes Reveals High Occurrence of Putative Parasitoids in the Plankton
Source: PLoS One. 2008 Jun 11;3(6):e2324. doi: 10.1371/journal.pone.0002324 (PMC2396521; doi:10.1371/journal.pone.0002324)

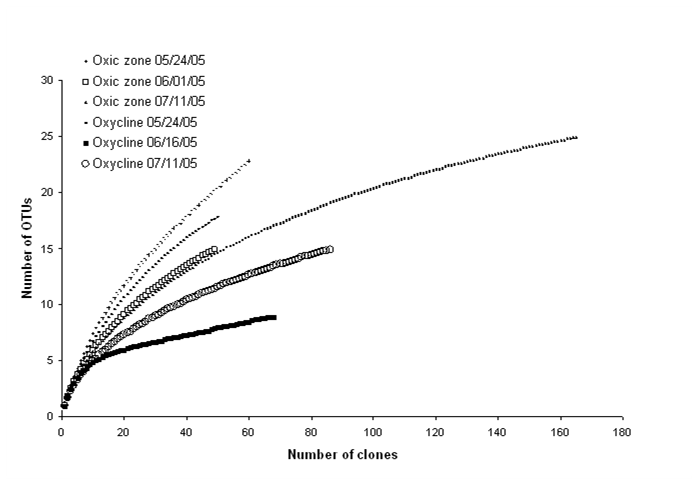

Supplement: Figure S1 — Rarefaction curves for six Lake Pavin libraries, spring/summer 2005. Clones were grouped into OTUs at a level of sequence similarity of ≥97% (0.07 MB DOC) [file pone.0002324.s003.tif]

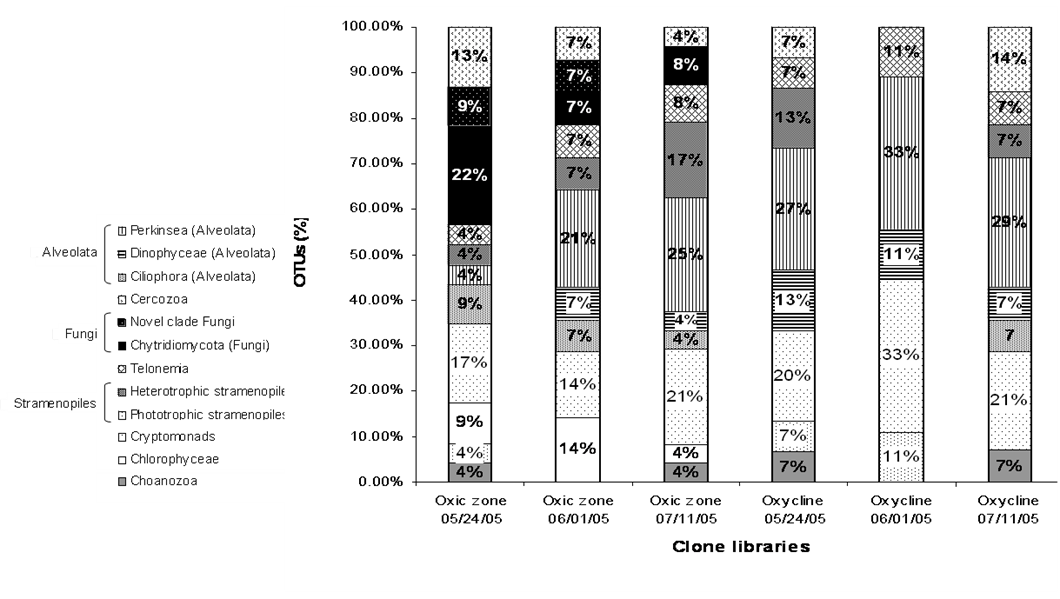

Supplement: Figure S2 — Relative abundances of OTUs within the 8 phylogenetic groups represented in our libraries from Lake Pavin, spring/summer 2005. Taxonomic subgroups are defined for the major taxa. The numerical abundances (i.e. % of total OTUs) are given for each subgroup (0.31 MB DOC) [file pone.0002324.s004.tif]
